# Genome-Wide Association Mapping of Acid Soil Resistance in Barley (*Hordeum vulgare* L.)

**DOI:** 10.3389/fpls.2016.00406

**Published:** 2016-03-31

**Authors:** Gaofeng Zhou, Sue Broughton, Xiao-Qi Zhang, Yanling Ma, Meixue Zhou, Chengdao Li

**Affiliations:** ^1^Western Barley Genetics Alliance, Department of Agriculture and Food, Government of Western Australia, PerthWA, Australia; ^2^Western Barley Genetics Alliance, Murdoch University, MurdochWA, Australia; ^3^TIA – Tasmanian Institute of Agriculture, University of Tasmania, Kings MeadowsTAS, Australia

**Keywords:** barley, acid soil resistance, aluminum resistance, association mapping, GWAS

## Abstract

Genome-wide association studies (GWAS) based on linkage disequilibrium (LD) have been used to detect QTLs underlying complex traits in major crops. In this study, we collected 218 barley (*Hordeum vulgare* L.) lines including wild barley and cultivated barley from China, Canada, Australia, and Europe. A total of 408 polymorphic markers were used for population structure and LD analysis. GWAS for acid soil resistance were performed on the population using a general linkage model (GLM) and a mixed linkage model (MLM), respectively. A total of 22 QTLs (quantitative trait loci) were detected with the GLM and MLM analyses. Two QTLs, close to markers bPb-1959 (133.1 cM) and bPb-8013 (86.7 cM), localized on chromosome 1H and 4H respectively, were consistently detected in two different trials with both the GLM and MLM analyses. Furthermore, bPb-8013, the closest marker to the major Al^3+^ resistance gene *HvAACT1* in barley, was identified to be QTL5. The QTLs could be used in marker-assisted selection to identify and pyramid different loci for improved acid soil resistance in barley.

## Introduction

Aluminum (Al) is the most abundant metal element in the earth’s crust. Al cations (particularly Al^3+^) are released from Al-containing compounds into soil solution at low pH. The soluble toxic Al^3+^ can rapidly inhibit root growth and influence nutrient and water uptake from acid soil, which explains why Al^3+^ is the major limiting factor affecting crop and pasture production in acid soils (Foy, 1983).

Around 30% of arable land in the world is acidic (von Uexkull and Mutert, 1995). Plant species vary widely in their ability to grow and yield on acid soils (Foy, 1983). Some species and even cultivars with in certain species have evolved mechanisms to adapt to toxic Al^3+^ in acid soils. Barley (*Hordeum vulgare* L.) is considered one of the most Al^3+^ sensitive cereal crops, and rice the most resistant (McLean and Gilbert, 1927). Two main mechanisms of resistance have been proposed: exclusion mechanism and resistance mechanism. The exclusion mechanism prevents Al^3+^ from entering cells and minimizes Al toxicity, while the resistance mechanism allows plants to take up Al^3+^ and accumulate Al^3+^ within their cells (Kochian et al., 2004).

The Al^3+^-induced secretion of organic acid anions from roots is the best example of exclusion mechanisms in higher plants, such as wheat (*Triticum aestivum*) and sorghum (*Sorghum bicolor*; Sasaki et al., 2004; Magalhaes et al., 2007; Ryan et al., 2009; Tovkach et al., 2013). Organic acids can chelate toxic Al^3+^ to form harmless complexes in the rhizosphere, thereby preventing Al^3+^ from damaging cellular components and resulting in detoxification of Al^3+^. In wheat, both malate and citrate secretion from roots has been associated with Al^3+^ resistance (Sasaki et al., 2004; Ryan et al., 2009), whereas in barley, only citrate exudation from roots has been identified (Furukawa et al., 2007). In addition, aluminum resistance between resistant wheat and barley was significantly different. For example, in nutrient solutions, Al^3+^ resistant wheat ‘ET8’ can grow well with over 90% of relative root length at 20 μM AlCl_3_, whilst the resistant barley ‘Dayton’ can only achieve this level of root growth in 2 μM AlCl_3_ (Sasaki et al., 2004; Zhou et al., 2013).

In addition to organic acid transporters, ABC transporters, and other proteins have also been reported to be associated with Al^3+^ resistance including: the C_2_H_2_-type Zn finger transcription factor gene *AtSTOP1* in *Arabidopsis* (Iuchi et al., 2007) and the *ART1* gene in rice (Yamaji et al., 2009), and the ABC transporter (UDP-glucose transporter) genes *STAR1* and *STAR2* in rice (Huang et al., 2009). Thus, a variety of transporters and proteins have been demonstrated to be involved in Al^3+^ resistance in plants.

Recently, Dai et al. (2013) identified 35 Al^3+^-associated proteins from wild barley which were involved in metabolism, cell growth, energy, protein storage, protein biosynthesis, signal transduction, and transporters, etc. There were four proteins specifically expressed in wild barley that were expressed in the Al^3+^ resistant cv. ‘Dayton.’ These results indicate that other mechanisms might also be involved in Al^3+^ resistance in barley and that it might be possible for us to discover new Al^3+^ resistance genes in barley.

Quantitative traits loci are commonly dissected with two tools, linkage analysis, and association mapping. Linkage analysis in plants is generally conducted using recombinant populations from bi-parental crosses, while association mapping typically examines the shared inheritance from individuals with unrelated ancestry. Great successes in identifying genes have been reported in humans (Michailidou et al., 2015) and plants (Yan et al., 2010) using association mapping strategies.

In the present study, we evaluated acid soil/Al^3+^ resistance in 218 barley accessions including wild barley from Tibetan and cultivated barley from all over the world. These lines were then used to investigate candidate QTLs for Al^3+^ resistance using association mapping approach.

## Materials and Methods

### Genotyping

Most Australian barley varieties and selected world wide barley cultivars based on their acid soil resistance and origin were used in the present study. The resistant and susceptible lines were included in the accessions. Leaves from 218 barley accessions were harvested and frozen below -80°C. Genomic DNA was extracted from each sample using the extraction method according to Stewart and Via (1993). Genomic representations and preparation of barley “discovery arrays” and “polymorphism-enriched arrays” were prepared as described by Wenzl et al. (2004). After DArT (Diversity Arrays Technology) genotyping, a quality parameter *Q* was calculated for each marker. The *Q* parameter is the variance of the hybridization intensity between allelic states as a percentage of the total variance. Those markers with a *Q* and call rate being both greater than 80% were selected for association mapping analysis. The DArT markers consensus genetic map was provided at http://www.diversityarrays.com.

### Evaluation of Al^3+^ resistance

Al^3+^ resistance was evaluated in Western Australia (WA) and Tasmania (TAS), respectively. In WA, acid soil with pH 4.2 was obtained from Merredin Research Station. Natural acid soil was collected from the 10 to 30 cm layer. Soil pH was 4.2 with soluble aluminum of 8.1 mg/kg. For the control treatment, lime was added to the same soil to adjust the pH to 6.5. Each pot (diameter 9 cm and height 22 cm) contained 1.2 kg soil. Water was added to maintain moisture at 90% of the field capacity. Seeds from each line were germinated in Petri dishes and seedlings with similar root lengths were planted in two pots with acid soil and two pots with limed soil (four seedlings per pot). The pots were randomly placed in a temperature-controlled glass house under a 16 h/8 h light/dark cycle (22 and 18°C, respectively). The longest root growth was measured 1 week after sowing and results were expressed as relativeroot length (RRL). RRL = *x*/*y* where *x* and *y* represent mean root length in acid and limed soil, respectively.

In TAS, acid soil with pH 4.3 was collected from Northern Tasmania. The exchangeable aluminum was 13.6 mg/kg. Acid soil was mixed well and then placed in a tank (length of 2 m, width of 1 m, and depth of 0.4 m) in a temperature-controlled glass house. Water level was controlled using an automatic watering system. Five seedlings of each line were randomly planted in the tank in November, 2013. Seeds were germinated in Petri dishes for 2 days and seedlings with similar root lengths were planted (3 cm distance) in the tank. The barley cultivar Golden Promise was planted in the buffer zone. Labels were placed every 10 seedlings. After 2 weeks, Al^3+^ resistance was scored based on root length. Resistance scores ranged from 0 (susceptible) to 11 (resistant). Control treatment was not conducted since our previous results showed that the absolute root length is also a better indication of Al^3+^ resistance with resistant lines always having root length of more than 8 cm while root length of sensitive ones being always less than 5 cm. The experiments were then repeated in May in 2014, and the method was the same to the first experiment in Tasmania. Two datasets were recorded as TAS1 (2013) and TAS2 (2014).

### Relationship between the Phenotyping Methods

The correlation coefficient (*r*) between the trials was calculated by the function CORREL in Excel. A hypothesis test (*t*-test) was used to evaluate whether or not a linear relationship existed between the two groups. 
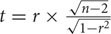
, where, *r* is the correlation coefficient and *n* is the number of barley accessions.

### Population Structure Analysis

The software Structure (version 2.3; Hubisz et al., 2009) was used to analyze the structure of these barley lines. Genotypes of the lines were imported to Structure. The length of burn in period was set to be 5,000, and the number of MCMC replications after burn in was set to be 5,000. The admixture model was used to conduct simulations. Simulations were conducted by running K (the number of populations) from 2 to 10. K was estimated as described by Evanno et al. (2005).

### Association Mapping

The software TASSEL (version 3.0) was used to conduct association mapping of acid soil resistance in barley. Information on genotype, genetic map, population structure, and traits were imported into Tassel 3.0. Kinship was estimated using genetic markers with Tassel 3.0. Association analysis for Al^3+^ resistance was carried out using both GLM and MLM analyses. The GLM model was: trait = population structure + marker effect + residual, while the MLM model is: trait = population structure + marker effect + individual + residual. Kinship was calculated with TASSEL 3.0. The association mapping results from the two models were compared. The significant threshold of *P-*values for assessing marker-trait-association (MTA) were calculated based on false discovery rate (FDR; Benjamini and Hochberg, 1995). The thresholds were determined after Bonferroni multiple test correction at a significant level of *P* = 0.05. The *P*-value from the *F*-test on markers was converted to -log10. The significant threshold was also used in the MLM analysis.

## Results

### Genotyping

A total of 1,157 DArT markers were scored in the barley population. The markers lacking chromosome position were removed. Among the remaining 482 markers, 408 markers with *P*-value (marker quality) above 80 were scored very reliably. These 408 DArT markers were used for structure analysis and LD analysis.

### Phenotyping

Acid soil resistance of 218 barley accessions were calculated for their correlation coefficient among these trials. The correlation coefficient between RL and RRL in WA trial was 0.84, and the correlation coefficient between TAS1 and TAS2 was 0.67. *t*-values of these two correlation coefficient, 24.9 and 14.5, were more than *t*_0.01,259_ = 2.57, indicating that RL and RRL in WA, and two experiments in TAS were linearly correlated. We then calculated the correlation coefficient between RRL in WA trial and mean phenotype in TAS trial. *r-*value was 0.56 between RRL in WA trial and phenotype in TAS trial. A hypothesis test (*t*-test) was used to evaluate whether a linear relationship exists between the two groups. The results showed that the *t*-value of correlation coefficient was 10.8, beingmore than *t*_0.01,259_ = 2.57, indicating that RRL in WA and TAS trial was linearly correlated. RRL in WA and two experiments in TAS were used to conduct association mapping analysis for Al^3+^ resistance.

### Population Structure

**FIGURE 1:**
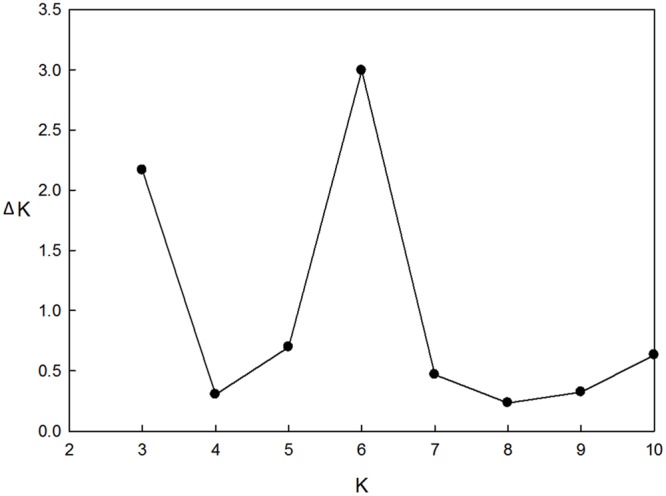
**Estimation of the most probable number of clusters (k), based on nine independent runs and k ranging from 2 to 10**.

**FIGURE 2:**
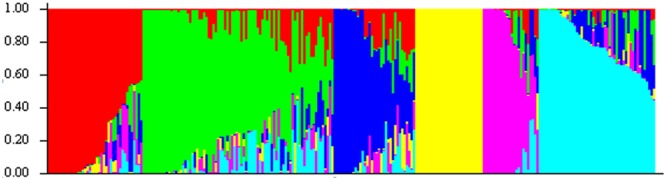
**Population structure of 218 barley accessions based on genetic diversity detected by 408 DArT markers with *k* = 6.** X axis represents 1∼218 accessions, and they were clustered in six groups. Each barley line ordered by membership coefficient (Q) is represented by a linepartitioned in colored segments that represent the individual’s estimated membership fractions.

These 218 barley accessions were used to analyze population structure. The cluster parameter *k* was set from 2 to 10. To determine the number of clusters suitable for association mapping analysis, the parameter Δk was applied. When *k* = 6, Δk reached a top value of ∼3.0 (**Figure 1**). According to the explanation of Evanno et al. (2005), the appropriate number of clusters should be six. The compositions of each cluster are shown in **Figure 2** and these clusters are represented by six different colors.

### Linkage Disequilibrium Decay

**FIGURE 3:**
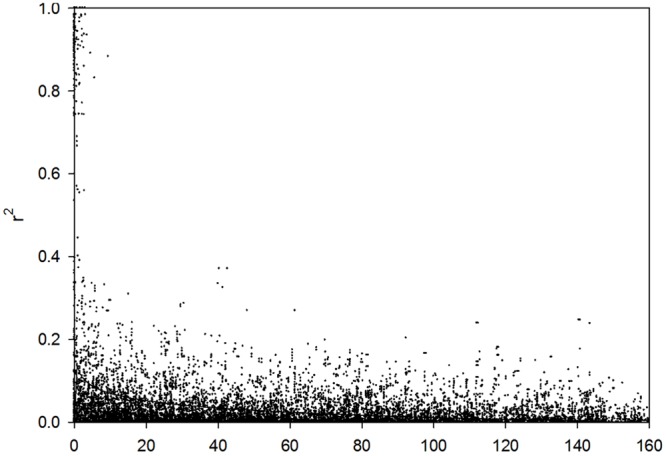
**Decay of LD of the whole barley genome**.

The LD decay of genetic distance in these 218 barley lines was 3.13 cM (*r*^2^ = 0.1; **Figure 3**). Therefore, the 408 DArT markers used in the present study will cover the entire barley genome and is sufficient for genome-wide association mapping analysis.

### QTL Controlling Al^3+^ Resistance in Barley

Three sets of Al^3+^-resistant phenotype data from WA and TAS were conducted using the GLM association mapping analysis. The significant level of threshold for the traits varied from 0.002 to 0.004. Their -log10 values were 2.4–2.7. As a compromise between these significant levels, an arbitrary threshold -log10 value of 2.5 was used for all experiments analysis. This threshold was also used in the MLM association mapping analysis.

**FIGURE 4:**
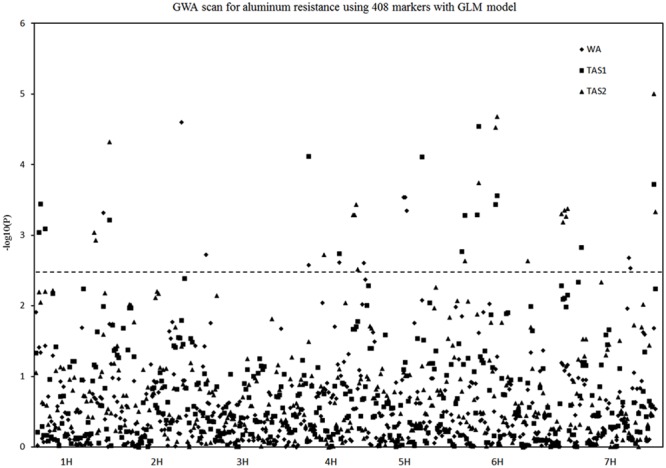
**Genome-wide association studies analysis of acid soil tolerance within 218 barley accessions with GLM.** Results from WA, TAS1, and TAS2 were shown with different shapes.

Twenty different QTLs were identified for acid soil resistance following the GLM analysis (**Figure 4** and **Table 1**). Eight QTLs for RRL from WA were identified from the WA data on chromosomes 1H, 2H, 3H, 4H, 5H, and 7H. Ten QTLs for acid soil resistance were identified from the TAS1 data and mapped to chromosomes 1H, 3H, 4H, 5H, 6H, and 7H. Ten QTLs were identified from the TAS2 data and mapped to chromosomes 1H, 4H, 5H, 6H, and 7H. Some QTLs were repeatedly mapped in two or three experiments. For example, the locus QTL1 (133–141 cM) on chromosome 1H was mapped in all three experiments, and QTL4, QTL5, QTL11, QTL12, QTL13, and QTL15 from chromosomes 3H, 4H, 6H, and 7H were detected in two experiments.

**Table 1:** Association mapping results for acid soil tolerance with the GLM analysis.

Trait	Marker	Chromosome (Chr.)	Position (cM)	-log10	Marker *R*^2^	Effect (A–B)	Loci
WA	bPb-1959	1H	133.1	3.3	0.075	-19.8	QTL1
WA	bPb-1815	2H	146.6	4.6	0.107	14.6	QTL2
WA	bPb-6048	2H	161.1	2.7	0.060	12.2	QTL3
WA	bPb-7684	3H	167.3	2.6	0.058	-13.6	QTL4
WA	bPb-8013	4H	86.7	2.6	0.058	12.4	QTL5
WA	bPb-0909	5H	45.6	2.6	0.057	10.7	QTL6
WA	bPb-4970	5H	139.0	3.5	0.083	-15.7	QTL7
WA	bPb-1420	5H	139.0	3.5	0.083	-15.7	QTL7
WA	bPb-4318	5H	139.0	3.3	0.077	-15.2	QTL7
WA	bPb-8539	7H	125.4	2.7	0.060	-11.3	QTL8
WA	bPb-1669	7H	125.4	2.5	0.056	-11.1	QTL8
TAS1	bPb-9608	1H	11.5	3.0	0.062	1.0	QTL9
TAS1	bPb-7137	1H	11.7	3.4	0.070	1.1	QTL9
TAS1	bPb-1318	1H	13.1	3.1	0.064	1.0	QTL9
TAS1	bPb-0395	1H	141.3	3.2	0.065	-1.3	QTL1
TAS1	bPb-7684	3H	167.3	4.1	0.087	-1.5	QTL4
TAS1	bPb-8013	4H	86.7	2.7	0.054	-1.1	QTL5
TAS1	bPb-1965	5H	171.9	4.1	0.083	1.2	QTL10
TAS1	bPb-9807	6H	38.0	3.3	0.066	0.9	QTL11
TAS1	bPb-2058	6H	38.0	2.8	0.054	1.0	QTL11
TAS1	bPb-2464	6H	63.8	3.3	0.066	-1.1	QTL12
TAS1	bPb-5822	6H	64.8	4.5	0.095	-1.4	QTL12
TAS1	bPb-5778	6H	84.6	3.6	0.073	-1.2	QTL13
TAS1	bPb-5903	6H	84.6	3.4	0.068	-1.1	QTL13
TAS1	bPb-0366	7H	58.0	2.8	0.058	-1.2	QTL14
TAS1	bPb-6701	7H	159.2	3.7	0.075	-1.6	QTL15
TAS2	bPb-7429	1H	106.2	3.0	0.068	1.4	QTL16
TAS2	bPb-9180	1H	106.2	2.9	0.064	1.4	QTL16
TAS2	bPb-0395	1H	141.3	4.3	0.099	-1.8	QTL1
TAS2	bPb-4990	4H	64.2	2.7	0.058	-1.7	QTL17
TAS2	bPb-9632	5H	31.0	3.3	0.072	1.1	QTL18
TAS2	bPb-6067	5H	31.0	3.4	0.076	1.2	QTL18
TAS2	bPb-0050	5H	31.0	3.3	0.072	1.1	QTL18
TAS2	bPb-2900	5H	31.8	2.5	0.054	1.0	QTL18
TAS2	bPb-9807	6H	38.0	2.6	0.057	0.9	QTL11
TAS2	bPb-5822	6H	64.8	3.7	0.087	-1.5	QTL12
TAS2	bPb-5778	6H	84.6	4.7	0.109	-1.6	QTL13
TAS2	bPb-5903	6H	84.6	4.5	0.103	-1.5	QTL13
TAS2	bPb-0108	7H	0.5	2.6	0.060	-1.0	QTL19
TAS2	bPb-2197	7H	30.3	3.4	0.077	1.3	QTL20
TAS2	bPb-8447	7H	30.3	3.4	0.076	1.3	QTL20
TAS2	bPb-7428	7H	30.3	3.3	0.073	1.3	QTL20
TAS2	bPb-6965	7H	30.3	3.3	0.074	1.3	QTL20
TAS2	bPb-4989	7H	30.3	3.2	0.070	1.3	QTL20
TAS2	bPb-6701	7H	159.2	5.0	0.113	-2.1	QTL15
TAS2	bPb-0375	7H	160.2	3.3	0.073	1.2	QTL15

**FIGURE 5:**
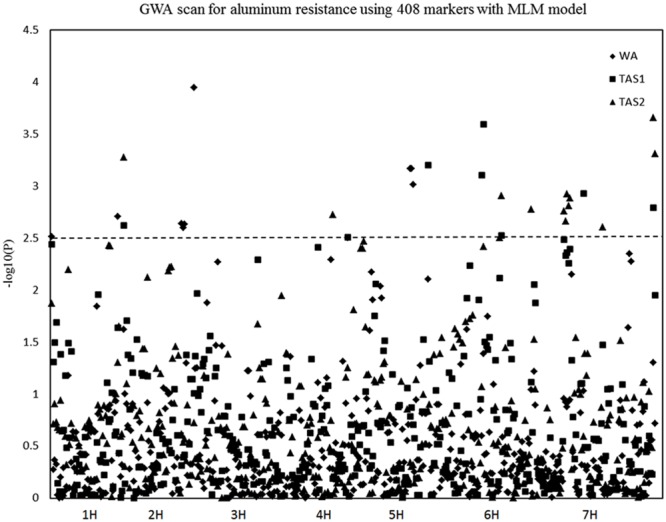
**Genome-wide association studies analysis of acid soil tolerance within 218 barley accessions with MLM.** Results from WA, TAS1, and TAS2 were shown with different shapes.

The MLM analysis was also used for association mapping analysis. A total of sixteen QTLs were identified for acid soil resistanceusing this analysis (**Figure 5** and **Table 2**). Among them, two new QTLs were identifieds from the MLM analysis and 14 QTLs overlapped with the results from the GLM analysis. The QTL3, QTL4, QTL6, QTL8, QTL11, and QTL16 were not detected with the MLM analysis. The QTL1 was also detected in all three experiments with the MLM analysis, and QTL5, QTL13, QTL15, and QTL20 were identifed in two experiments (WA and TAS1 or TAS1 and TAS2).

**Table 2:** Association mapping results for acid soil tolerance with the MLM analysis.

Trait	Marker	Chromosome (Chr.)	Position (cM)	-log10	Marker *R*^2^	Effect (A–B)	Loci
WA	bPb-6451	1H	3.3	2.5	0.062	-9.8	QTL9
WA	bPb-1959	1H	133.1	2.7	0.064	-18.9	QTL1
WA	bPb-3925	2H	131.5	2.6	0.062	-11.5	QTL21
WA	bPb-8302	2H	131.5	2.6	0.068	-11.6	QTL21
WA	bPb-1103	2H	131.5	2.6	0.061	-11.5	QTL21
WA	bPb-1815	2H	146.6	3.9	0.101	12.9	QTL2
WA	bPb-8013	4H	86.7	2.5	0.056	11.1	QTL5
WA	bPb-4970	5H	139.0	3.2	0.079	-12.9	QTL7
WA	bPb-1420	5H	139.0	3.2	0.079	-12.9	QTL7
WA	bPb-4318	5H	139.0	3.0	0.074	-12.6	QTL7
TAS1	bPb-0395	1H	141.3	2.6	0.051	-1.2	QTL1
TAS1	bPb-8013	4H	86.7	2.5	0.048	-0.9	QTL5
TAS1	bPb-1965	5H	171.9	3.2	0.064	0.9	QTL10
TAS1	bPb-2464	6H	63.8	3.1	0.063	-1.0	QTL12
TAS1	bPb-5822	6H	64.8	3.6	0.076	-1.2	QTL12
TAS1	bPb-5778	6H	84.6	2.5	0.050	-0.9	QTL13
TAS1	bPb-7428	7H	30.3	2.5	0.047	0.9	QTL20
TAS1	bPb-0366	7H	58.0	2.9	0.060	-1.0	QTL14
TAS1	bPb-6701	7H	159.2	2.8	0.054	-1.5	QTL15
TAS2	bPb-0395	1H	141.3	3.3	0.075	-1.5	QTL1
TAS2	bPb-4990	4H	64.2	2.7	0.059	-1.6	QTL17
TAS2	bPb-6067	5H	31.0	2.5	0.053	1.0	QTL18
TAS2	bPb-5778	6H	84.6	2.9	0.065	-1.2	QTL13
TAS2	bPb-5903	6H	84.6	2.5	0.054	-1.0	QTL13
TAS2	bPb-0108	7H	0.5	2.8	0.069	-1.0	QTL19
TAS2	bPb-8447	7H	30.3	2.9	0.066	1.2	QTL20
TAS2	bPb-2197	7H	30.3	2.9	0.064	1.2	QTL20
TAS2	bPb-6965	7H	30.3	2.8	0.062	1.1	QTL20
TAS2	bPb-7428	7H	30.3	2.8	0.060	1.1	QTL20
TAS2	bPb-4989	7H	30.3	2.7	0.058	1.1	QTL20
TAS2	bPb-7517	7H	94.2	2.6	0.057	1.7	QTL22
TAS2	bPb-6701	7H	159.2	3.7	0.085	-1.9	QTL15
TAS2	bPb-0375	7H	160.2	3.3	0.076	1.1	QTL15

In summary, 16 of the same QTLs were detected in both the GLM and MLM analysis. Six QTLs were only identified with the GLM analysis, and two QTLs (QTL21 and QTL22) were only mapped with the MLM analysis. For RRL from WA, the same four QTLs were identified with both analysis methods. For the TAS1 resistance score, seven QTLs were detected with both analysis methods, and the seven same QTLs were also mapped for TAS2 data with both the GLM and MLM analyses.

### Anchoring Al^3+^ Resistance Gene *HvAACT1*

The *HvAACT1* gene (accession No. AB302223.1) sequence was blasted with barley cv. Morex genomic sequences (http://webblast.ipk-gatersleben.de/barley/). The gene was anchored to Morex_contig_51279, and the genetic position was 60.55 cM on chromosome 4H based on the Morex consensus map. The QTL5 marker bPb-8013 on chromosome 4H was first anchored to Barke_contig_278219, then its end sequences were mapped to Morex_contig_107862. In the Morex consensus map, its position was 68.98 cM on chromosome 4H. The marker bPb-8013 was 8.43 cM away from the *HvAACT1* gene. We checked marker-density in the region, and found that the closest marker to bPb-8103 was bPb-6949 (72.2 cM). The marker bPb-6949 was directly anchored to Morex_contig_38805 and Bowman_contig_94924, but the genetic position was not provided. By blasting Bowman_contig_94924 end sequences, we were able to map bPb-6949 into Morex_contig_43905, at 51.4 cM on chromosome 4H. Therefore, bPb-8013 was the closest marker to the *HvAACT1* gene in the present study.

### Elite Al^3+^ Resistant Lines

Based on Al^3+^ resistance in WA and TAS, 49 barley lines with promising Al^3+^ resistance were identified in **Table 3**. Among these 49 lines, HOR 8847, B1079, and 115-9505-B showed excellent Al^3+^ resistance in both the WA and TAS trials. The RRL of these three lines ranged from 102.4 to 134.7% in WA, and their Al^3+^ resistance scores in TAS ranged from 7.0 to 10.5.

**Table 3:** Elite Al^3+^ resistant barley lines based on their roots growth.

	RRL in WA (%)	Al tolerance TAS1	Al tolerance TAS2
HOR 8847	105.1	8.0	10.5
B1079	134.7	7.5	8.5
115-9505-B	102.4	7.0	9.5
HOR 8849	87.4	8.5	9.0
BR 1	105.5	7.5	–
Spanish landrace 338c	79.2	8.0	10.5
Carmen	106.2	8.0	6.0
B1121	93.7	6.0	8.5
FM404	84.2	7.5	8.0
Oram 257-3	97.5	8.5	5.5
Spanish landrace 349b	80.6	8.0	7.5
Oram 257-1	88.4	6.5	7.0
Spanish landrace 336d	68.7	7.5	9.5
HOR 8846	66.6	8.5	11.0
Spanish landrace 349	68.9	7.5	9.0
116-9707-B	72.5	8.0	7.5
Yiwu Erleng	93.3	5.5	6.0
Carmen-B	86.3	6.5	6.0
Dayton	91.9	5.5	6.0
KAJSA	79.0	5.5	7.5
HOR 8850	69.3	7.5	7.5
YRJAR	73.4	6.5	7.0
Aurora	66.6	7.5	8.0
Rosa	74.1	5.5	7.5
HOR 8848	61.9	8.5	7.5
JSELM	77.6	4.5	6.5
B1100	87.4	4.0	5.5
WA 12925	68.3	4.5	7.0
Oram 385-2-2	55.9	7.0	8.5
B1043	74.2	4.0	5.5
B1118	65.8	4.5	7.0
YYXT	71.4	3.0	7.0
HOR 12820	67.2	4.5	6.0
Macquarie	59.1	7.0	6.5
Lang/Carmen-B	66.6	6.5	4.5
Oram 258-3	64.2	6.5	5.0
Fischers Wirchen blatter 2	60.6	6.5	5.5
Hindmarsh	62.6	5.5	6.0
TF026	71.6	4.0	5.0
B1064	65.4	4.0	6.5
WA 12944	56.4	6.0	6.5
YUQS	56.4	4.5	8.0
B1052	64.5	5.0	4.5
WA 12937	60.5	5.0	5.5
HOR 4052	62.2	4.5	5.0
WA 12914	59.4	3.5	7.0
Kombainiesis	60.3	5.0	5.0
HOR 3870	60.4	5.5	3.5
Boa Fe	55.1	5.0	4.5

## Discussion and Conclusion

### GWS Results as Affected by Models and Evaluation Method

Both GLM and MLM methods were used for association mapping analysis in barley according to previous studies (Mohammadi et al., 2014; Zhou H. et al., 2014; Gutierrez et al., 2015; Tamang et al., 2015). Zhou H. et al., 2014 used the MLM analysis to detect QTLs for stem rust resistance in US barley germplasm. Tamang et al. (2015) used both the GLM and MLM methods to conduct association mapping analysis for spot form net blotch in barley. In the present study, we carried out association mapping analysis with both the GLM and MLM analyses. Twenty QTLs for aluminum resistance were identified with the GLM analysis, while among these 20 QTLs, 14 QTLs were detected with MLM model. In addition, two new QTLs were mapped with the MLM analysis. However, the strength of association with the MLM analysis was weaker, that six QTLs detected by GLM did not pass the Bonferroni multiple test threshold with the MLM analysis. Additionally, the two new QTLs (QTL21 and TQL22) detected with the MLM method, only just passed the Bonferroni multiple test threshold with their -log10 *P*-values of ∼2.6.

The results from the GLM and MLM analyses were compared for each experiment. Eight QTLs were found for RRL (WA experiment) with the GLM analysis, whereas, only four of the same QTLs (QTL1, QTL2, QTL5, and QTL7) were identified with the MLM analysis. For the TAS1 experiment, 7 out of 10 QTLs mapped with the GLM method were also detected with the MLM method. The same trend was also found in the association mapping analysis of the TAS2 data, where seven QTLs were detected with both the GLM and MLM analysis.

### Association Mapping Results as Affected by Different Acid Soils

Many studies assessed barley Al^3+^ resistance by measuring longest root length (LRL) and relative longest root length (RLRL) in acid soils (Cai et al., 2013; Zhou et al., 2013; Zhou H. et al., 2014). They found that RL, LRL, and RLRL could be used to investigate the acid soil resistance in plants. These traits were positively correlated. In this study, we evaluated acid soil resistance by RRL and a root length resistance score. We attempted to map all QTLs responsible for Al^3+^ resistance in acid soil.

Two acid soils were used in the present study. In the WA acid soil experiment, barley resistance was assessed by RRL, whereas, in the TAS1 and TAS2 soil experiments, barley germplasm resistance was evaluated using a comparative score of root length. Using the GLM analysis, only three QTLs (QTL1, QTL4, and QTL5) were detected in both WA and TAS1 or TAS2 experiments. When they were analyzed with the MLM method, only two QTLs (QTL1 and QTL5) were identified in both WA and TAS1 or TAS2 experiments.

The results showed that only QTL1 and QTL5 were detected for acid soil resistance in both the WA and TAS experiments with both the GLM and MLM analysis methods. The QTL5 marker bPb-8013 (4H) is close to the barley Al^3+^ resistance gene *HvAACT1* which encodes a citrate transporter (Furukawa et al., 2007; Zhou et al., 2013). This indicates that the association mapping method was successful in detecting acid soil resistance genes.

There were several reasons which may explain a great proportion of the different QTLs for acid soil resistance between the WA and TAS experiments. Firstly, the acid soils used in WA and TAS experiments were from different locations, which lead to the possible difference in the composition and nutrient status of the soils. Root length is influenced by Al^3+^ as well as many other ions. Secondly, acid soil resistance was assessed by RRL in WA, taking into account root growth in both acid and limed soil. In contrast, root length was evaluated only in acid soil in the TAS experiments, so some QTLs may be associated with root growth vigor.

**Table 2** showed that QTLs for Al^3+^ resistance were detected on chromosomes 1H, 4H, 5H, 6H, and 7H in both TAS1 and TAS2 experiments. Among these QTLs, four QTLs (QTL1, QTL13, QTL15, and QTL20) were identified in both TAS experiments. Both the distance between QTL5 and QTL17 on chromosome 4H, and the distance between QTL12 and QTL13 on chromosome 6H were ∼20 cM away. For chromosomes 5H and 7H QTLs, some QTLs were identified in different positions. The QTLs differences between TAS1 and TAS2 may be caused by several reasons, including light length difference between May and November, and seed growth vigor difference caused by seed storage. Furthermore, the trait for acid soil resistance is a quantitative trait, and slight phenotype differences may influence the detection of minor QTLs.

### Genes and QTLs for Al^3+^ Resistance in Barley

Map-based cloning has been used in barley to clone the Al^3+^ resistance gene *HvAACT1*, which encodes a citrate transporter (Furukawa et al., 2007). However, with limited genetic diversity of parental lines used in previous QTL mapping studies (Raman et al., 2002), only Al^3+^ resistance genes which show diversity between parental lines in mapping populations could be detected. In other plant species, researchers have found that other mechanisms are also involved in Al^3+^ resistance, including the C_2_H_2_-type Zn finger transcription factor AtSTOP1 in *Arabidopsis* (Iuchi et al., 2007), the ART1 in rice (Yamaji et al., 2009), and the ABC transporter (UDP-glucose transporter) STAR1 and STAR2 in rice (Huang et al., 2009). Recently, Delhaize et al. (2012) reported that Al^3+^ tolerance of root hairs in wheat was encoded by genes independent of the *TaALMT1* gene. Whether, similar mechanisms except citrate transporters in other species involved in barley Al^3+^ resistance needs further investigation.

Genome-wide association mapping uses a natural population instead of a recombinant population from two parental lines. Natural populations often contain a greater variety of genetic diversity. Using GWAS techniques, it is feasible to identify putative QTLs for acid soil resistance across the barley whole genome. A similar research has been reported by Cai et al. (2013), but a different method was used to evaluate Al^3+^ resistance. Cai et al. (2013) used hydroponic methods to assess Al^3+^ resistance whilst we evaluated Al^3+^ resistance in acid soils in the present study. There are similarities across methods as eight out of the 22 QTLs identified in our study were also reported by Cai et al. (2013). Most notably, the novel Tibetan group-specific loci bPb-8524 (58.02 cM) and the Al^3+^ resistance gene *HvAACT1* marker bPb-6949 (72.21 cM), on chromosome 2H and 4H respectively, were mapped in the same region by Cai et al. (2013) and the present study.

It is well-known that barley cultivar Dayton carrying *HvAACT1* gene is Al^3+^-resistant and has been used for a positive control line in Al^3+^ resistance studies (Zhou et al., 2013; Zhou G. et al., 2014). In the present study, 49 most Al^3+^-resistant barley lines including Dayton were identified (**Table 3**) and the QTLs controlling Al^3+^ resistance in these lines were identified. The Al^3+^ resistant lines not carrying the *HvAACT1* gene will be selected to explore different mechanisms and conduct fine mapping studies. In addition, by investigating the different QTLs among these lines, we can pyramid different Al^3+^ resistant loci to breed more resistant lines.

## Conclusion

Twenty-two QTLs for Al^3+^ resistance were identified across barley genome, and these QTLs provide an insight into the genetic architecture of Al^3+^resistance in barley. The markers can be used for marker-assisted selection in barley breeding projects. *HvAACT1* gene has been well-studied in barley, whilst other QTLs underlying acid soil resistance are still unknown. Further, work remains to develop recombinant populations from barley lines carrying different Al^3+^ resistance loci, to facilitate fine mapping and map-based cloning of Al^3+^ resistance genes in barley.

## Author Contributions

CL and MZ designed the work; SB, X-QZ, YM prepared the barley germplasm and did genotyping and phenotyping; GZ analyzed and interpreted the data; GZ drafted the paper; all authors revised the paper and approved the final version to be published.

## Conflict of Interest Statement

The authors declare that the research was conducted in the absence of any commercial or financial relationships that could be construed as a potential conflict of interest.

The reviewer GZ declared a past co-authorship with one of the authors X-QZ and CL to the handling Editor, who ensured that the process met the standards of a fair and objective review.

## Abbreviations

Al: aluminum
GLM: general linkage model
GWAS: genome-wide association studies
LD: linkage disequilibrium
MLM: mixed linkage model
QTL: quantitative trait loci
